# ‘Genes versus children’: if the goal is parenthood, are we using the optimal approach?

**DOI:** 10.1093/humrep/dez256

**Published:** 2020-01-09

**Authors:** Jackson C Kirkman-Brown, Mariana V Martins

**Affiliations:** 1 Centre for Human Reproductive Science, IMSR, College of Medical and Dental Sciences, University of Birmingham, Birmingham B15 2TT, UK; 2 Birmingham Women’s Fertility Centre, Birmingham Women’s & Children’s NHS Foundation Trust, Birmingham B15 2TG, UK; 3 Faculty of Psychology and Educational Sciences, University of Porto, Porto 4200-135, Portugal; 4 Centre for Psychology at University of Porto, Porto 4200-135, Portugal

**Keywords:** ART, donation, age-related fertility, fertility counselling, genetics

## Abstract

First medical contact for couples trying for a child will usually emphasise the array of assistance available to ‘help them have their own child’, usually with options involving ART, after diagnosis. For many poorer prognosis couples, this means repetitive unsuccessful cycles of invasive and stressful treatment. What is sometimes lost at this stage is a reflection on the likelihood of success of different options, which may lead patients to focus on hoping for their own ‘genetic’ progeny, but failing to consider the alternative and potentially more successful other options, including donation and adoption, for achieving parenthood of a child. Factors not only such as female age but also advanced requirements such as preimplantation genetic testing or even mitochondrial replacement therapies all have reduced chances of success but further tend to reinforce the importance of a genetic link. The financial, physical and psychosocial burden associated with cumulative failure also lead to a higher probability of dropout and consequently an even higher probability of remaining in involuntary childlessness. We advocate formulation of a detailed roadmap for discussion of parenthood, with reference explanation to genetics and epigenetics, which gives due consideration to the psychological effects from the beginning to end of the treatment process, alongside a balanced consideration of the likelihood of treatment success and discussion of other options. Only when we provide patients with the service of a clear and transparent discussion of these matters, we will really realise the true potential of our field, which may then be better considered as assisted families.

## Introduction

A conception on 10 November 1977, followed by the healthy birth of Louise Brown on 25 July 1978, marked the start of an explosion in assisted conception provision and a generation of hope for subfertile couples. Before IVF, there were three main simple choices: acceptance of childlessness; adoption; or when the problem was male, using donor sperm. IVF changed this paradigm, not only by adding a new option but also by providing a new hope. Since 1978, these options have proliferated. Notable developments that have increased the chance of having their own genetic children include ICSI, reducing the need for sperm donation (Palermo *et al.*, 1992); pre-implantation diagnostic testing (Handyside *et al.*, 1992) and most recently mitochondrial replacement techniques (MRTs; Zhang *et al.*, 2017). The potential importance of a child being parents’ ‘genetically own’ is also further emphasised by developments in sperm and egg freezing, including social egg freezing, gonadal tissue preservation and stem cell-generated gametes, the latter being touted as just over the horizon. With such a wide range of techniques and science being discussed and portrayed in the media, the non-specialist could be forgiven for thinking the age where everyone can have their own genetic progeny is upon us. In fact, the reality is that for many couples, assisted conception techniques are often not particularly successful, and the chance of ‘own genes’ success may be rather low or zero. Equally, some of the more complex techniques (e.g. MRT, oocyte activation or stem cells) bear an unknown and unquantifiable risk to the potential child and future generations.

Concurrently, we have been reminded that the diagnosis of infertility is somewhat arbitrary, and both clinicians and patients like to go on with treatment even in the case of unproven therapies (Evers, 2016). For example, despite the fact that expectant management should be prescribed to younger couples with good prognosis (Donckers *et al.*, 2011), one-third is overtreated (Kersten *et al.*, 2014). There are, however, no studies on overtreatment of couples with poor prognosis. This seems of particular importance considering, first, the success rates of using donation and, second, the role of age-related fertility and the fact that older couples (female age ≥ 40 years) seek fertility treatment over six times more frequently than younger ones (de Graaff *et al.*, 2011).

In a situation where finances and emotions are usually both limiting, it may be time to re-examine our eagerness to reproduce our personal genes. Our coding genes may be sufficiently indistinct from another potential gamete donor that considering them so selfish (in the Richard Dawkins’ sense) that they are all that matters in parenthood is misguided. It is also of note that in 21^st^ century life, ‘families’ and morality around progeny have changed beyond recognition, so this concept of needing to pass on our own genes (for inheritance or future security) is perhaps outdated in the developed world; equally in African cultures, many women (unlike their partners) may have a strong need for a child even though it need not necessarily be their own offspring (Gerrits and Shaw, 2010). Still, there is a societal significance tied to the idea of having a genetically related child (Segers *et al.*, 2019). The present article advocates addressing possible concerns about donor conception for poorer prognosis patients from the time of diagnosis to potentially prevent psychological suffering, dropout and involuntary childlessness.

## Prognosis and chances of success

Putting genetic inheritance aside, we would be advising third-party reproduction right from the beginning in cases of poor prognosis and would be performing these techniques much sooner. Although most societies and authorities recommend oocyte donation when previous IVF cycles fail, there is no mention as to what a reasonable number of failed cycles should be.

Live birth rate (LBR) per embryo transfer with egg or embryo donation is above 50%, and the odds of a live birth (LB) are significantly higher in donor IVF cycles than autologous cycles (odds ratio: 1.26, 95% confidence interval: 1.18–1.33, adjusting for patient age, number of oocytes retrieved, and number of embryos transferred) (Yeh *et al.*, 2014). The odds of not having a LB decrease not only with the use of own oocytes and female age but also with treatment history and each ART cycle (Templeton *et al.*, 1996; Nelson and Lawlor, 2011; Vaegter *et al.*, 2017). This means that we might be contributing to unnecessary burden in some couples, repeating cycles with their own gametes that should be, sooner rather than later, transitioned to donation. This subject is even more important if we consider that reproductive health professionals have difficulties in guiding patients with poor prognosis to use donated gametes in the face of the high hopes and unrealistic expectations they show (Klitzman, 2016).

After an unsuccessful cycle, couples often face difficult decisions around continuation or cessation of treatment (Mesquita da Silva *et al.*, 2018) and, even in a situation independent of financial constraints, many couples are noted as discontinuing treatment due to the psychological burden and lost hope of success (Lande *et al.*, 2015). With all but the most severe (zero-chance) prognoses, once started on an own-genes route couples will often feel the need to pursue this, potentially enticed by hope associated with patient-friendly offers of advanced scientific technologies and interventions.

A key question to answer may be ‘are patients on the wrong path when the real problem is being childless?’ There have been many detailed discussions of childlessness (e.g. Kreyenfeld, 2018). Of note, the study of Vikstrom *et al.* (2015) found few mental health problems in women who had undergone IVF 20 years before compared to a reference group, but found increased levels of depression and phobic anxiety in all women who remained childless compared to those who had since become mothers. Having an unfulfilled child-wish after fertility treatment is associated with worse mental health for women even if they already had children (Gameiro *et al.*, 2014). For a 40-year-old European woman with average height and weight (primary infertility, not diagnosed), the probability of this wish to be fulfilled with her own eggs is 23%, against 55% if she opts for oocyte donation (Luke *et al.*, 2014). ART providers should also keep in mind that around one in four patients discontinue treatment (Brandes *et al.*, 2011; Pedro *et al.*, 2017); it is then perhaps tempting to consider for these couples that a much earlier discussion of alternatives may have helped their treatment journey.

## Understanding genomes and heritability

As genomic ancestry technologies have gained widespread availability, individuals have tended to become more aware of the concept of their personal genome. However, there may be a lack of awareness that in terms of coding genes, those we think of as ‘creating’ a child, over half are in common with even plants and most are in common with animal species. When we look at human genome variation, current data suggests that individuals may have >4 million differences compared with the reference genome, with ~2000 of these associated with complex traits, >25 of which may have negative disease implications (Sudmant *et al.*, 2015). This sounds like an incredible amount of unique difference, but the relevance for the random mix of 50% of these differences, both positive and negative, (with those from the other gamete) means that to consider the coding genes alone as the key piece of parental inheritance may be considered somewhat misguided. Patients may also have concepts that attribute more properties to these genetic variants, in terms of the day-to-day physiology and psychology of their child, than evidence supports. This is separate to those couples with genetic-based fertility issues, such as Y-deletions or recessive genetic disorders, who still wish to pass these along rather than consider ‘non-own genes’ children.

The astounding recent advances in genomics are also influencing the public opinion to be more pro ‘nature’ in the debate of nature versus nurture (Plomin, 2013). Recipient candidates for third-party reproduction often inquire about the influence of genetics and ask if donors were screened for cognitive and personality traits such as intelligence or neuroticism. Though heritability estimates are in general between 30 and 50% regarding personality (Plomin and Deary, 2015), it has now been well established that experiences and the environment influence the expression of personality (Briley and Tucker-Drob, 2014; Baumert *et al.*, 2017). There is now also solid evidence that while heritability of personality traits decreases over the lifespan, environmental influence increases (Wrzus and Roberts, 2017). In the end, the way that each individual perceives their experiences and rates them in terms of its relevance and influence is what makes siblings very different. In a process with an increasing sense of loss of control, patients seem to forget that one of the goals of parenting is the socialisation of personality. Sharing this kind of information might be important to address some patient concerns such as the presumption that there is a special bond between gamete donor and child (Goedeke *et al.*, 2015).

Whereas almost all of our coding genes may be the same, whomsoever they came from, the evolving field of epigenetics is revealing that non-genetic code effects can radically affect child health. This is perhaps most interesting in new understandings of epigenetics where we now know that the gestational mother’s lifestyle behaviour and nutritional metabolic health have direct effects on the offspring; arguably larger than those of any generalised developmental genetic predisposition chance, as described above. This knowledge of the importance of gestation is often neglected in early discussions with women but may be key in decision-making and acceptance when prospectively choosing own over donor-oocyte treatment.

## Risk to a child

Our discussion so far has focused upon the potential consequences and thoughts related to childlessness on an individual. However, we should also not fail to consider the potential consequences to succeeding generations of offspring in pursuing an own-genes approach. Indeed, with 5% of Northern European children conceived by ART, approaches that may increase the likelihood of unhealthy individuals also become a public health issue. Couples sometimes perceive potential outcomes of a lack of genetic link as giving rise to physically or psychologically unhealthy child (Eisenberg *et al.*, 2010), but conversely, there are no reports of concerns with using own-genes ‘old eggs’ or ‘damaged sperm’. Age of both mothers and fathers at time of conception is associated with an increasing number of genetic mutations carried by children and with increasing oxidative stress, which in turn is associated with neurological diseases and childhood cancers (Xavier *et al.*, 2019). From ICSI to artificial oocyte activation by ionophore or MRT, the fast and some what unchecked-pace of progress in assisted conception has seen techniques emerge with even simple safety-to-offspring data following on twenty or more years behind. ICSI using the father’s sperm is associated with a higher risk of birth defects than ICSI with donor sperm, and ICSI-conceived males appear to inherit the deficient spermatogenesis of their fathers (Berntsen *et al.*, 2019). Indeed, studies of children born by ART generally show higher perinatal and child health risks than for those who are spontaneously conceived (Berntsen, *et al.*, 2019). In the case of MRT or *in vitro* gametogenesis from stem cells, it seems certain that potential risks to offspring and succeeding generations are higher than natural or donor conception, but this remains barely discussed in the face of an own-genes demand and highly publicised scientific endeavours. Equally there should be caution that a eugenic approach of always using an ‘ideal’ donor is not started; for good prognosis couples, ART works well and produces many healthy children, these rates would indeed likely be even higher if those of poorer prognosis were not included. When treatment success rates for individuals would undoubtedly be higher and of clearer safety with donor gametes or indeed where adoption could be considered 100% successful in having a child to raise, when and where should clinicians encourage couples to consider the safety of their offspring in treatment choices? This needs urgent consideration as the use of these treatments is here now. It is notable that regulators often decide to allow novel treatments even when scant safety data is available (e.g. in UK, the Human Fertilisation and Embryology Authority allows oocyte activation and MRT): this can not only be judged as supporting patient choice but may also be providing further fuel to the concept of own-genes being of prime importance in parenting.

## Long-term psychosocial consequences of donor gamete use

Disclosure to the future child about their mode of conception has been probably the most studied and controversial subject when it comes to decision-making to pursue this course of treatment. Although it has been widely advised due to the potential psychological harms of secrecy and underestimation of medical issues, less than half of parents with children aged 10 years and older report having disclosed to them (Zanchettin *et al.*, 2016). Parents who opted for anonymity have shared apprehensions about health and accidental contact between donor-siblings (Nelson *et al.*, 2015). With the guarantee of anonymity no longer being a possibility in the near future due to direct-to-consumer DNA testing (Harper *et al.*, 2016) and increasing legal frameworks of openness (including retrospective legislation), we expect the scenario to change.

Prospective parents have concerns about how they will be perceived as parents without a genetic tie, and these concerns can remain in the absence of resemblance to the child (Isaksson *et al.*, 2019). However, the anxiety underlying the lack of biological ties decreases after birth, and confidence about the importance of socialisation increases (Indekeu *et al.*,2014). In qualitative studies, donor recipients use descriptions like ‘doing parenthood’ (Nordqvist & Smart, 2014) or ‘resemblance through nurture’ (Isaksson *et al.*, 2019).

We now have some evidence that both donor-conceived children (from early childhood to adolescence) and their parents are psychologically well adjusted and do not differ from families with spontaneously or own-gamete conceived children (e.g. Golombok *et al.*, 2011; Golombok *et al.*, 2013; Golombok *et al.*, 2017). High relationship quality has also been shown between mother and infant, with similar representations between both genetically related fathers and non-related (Golombok *et al.*, 2005; Imrie *et al.*, 2018).

Early disclosure therefore does not seem to pose difficulties in terms of psychological adjustment. In a qualitative study involving 44 adolescents whose parents used third-party ART and disclosed the mode of conception, Zadeh *et al.* (2018) found that the vast majority (36) felt indifferent about their conception. A prospective study suggests that amongst adolescents who were told about their biological origins, those who knew before the age of 7 years had higher psychological adjustment and family relationship quality (Ilioi *et al.*, 2017). There has been considerable questioning and debate around the benefits of disclosure versus non-disclosure (e.g. Crawshaw *et al.*, 2017; Golombok, 2017; Pennings, 2017a, 2017b), with research being still scarce and insufficient. The available evidence suggests that there is no deleterious effect of secrecy when comparing adolescents who were not told with those who were told (Kovacs *et al.*, 2015; Ilioi *et al.* 2017). There are, however, narratives of shock and disbelief in adults who found out that they were donor-conceived at adolescence or adulthood (Frith *et al.*, 2018). These can become more intricate if donation occurred within an anonymous legislation system and when the discovery is made through direct-to-consumer DNA testing, including the finding of siblings (Crawshaw, 2018). It is also curious to notice that in couples who were followed up for 10 years, the divorce rate of couples who had used gamete donation was not significantly different from those who had conceived naturally (Blake *et al.*, 2012). This is especially interesting if we consider that in a study of over 40 000 ART patients compared with an age-matched control group from the general population, the risk of divorce was attributed to childlessness, regardless of having gone through ART (Martins *et al.*, 2018). Again, it might be worth taking a step back and putting the emphasis on becoming a family over genetic relatedness.

The path of gamete donation is certainly not an option for everyone, and there are patients who discard this possibility from the start. For those who might be open to it, we therefore contest that information regarding the success rates and exploration of the misconceptions and myths involved should be included from the earliest stages of the diagnostic and treatment pathway in order to guide couples.

## Good decisions with good information

At present, exploring the preconceptions and informing on the legal, psychological and social implications of having a family when using donated genetic material is the keystone of fertility counselling and is recommended by the leading societies and regulators. However, opting for gamete donation has been described as a process that starts after many years of trying to conceive and unsuccessful treatment, with recommendations of referral to a fertility counsellor in order to come to terms with the decision (Greenfeld, 2015). Moreover, for many counsellors, this still seems to be viewed as part of a process where the focus on alternative ways of starting a family must be preceded by grieving the loss of genetic parenthood and focus on the impact and acceptance of infertility. We believe that the emphasis on this being a grieving process may again over-emphasise to the individual a medico-societal level of importance placed on their genetics.

A recent systematic review that included over 27 000 patients undergoing treatments, screenings or tests of any sort concluded that the majority of participants overestimated the intervention benefits and underestimated harm (Hoffmann and Del Mar, 2015). It is well known that ART patients especially tend to overestimate and have excessive confidence in the success of fertility treatment (Kamphuis *et al.*, 2014). In addition, patients might be using time-to-pregnancy as the preferred outcome instead of LB, which can be related to evidence showing that immediate health benefits are perceived as more important to patients than long-term ones (Albertini *et al.*, 2017). Moreover, it is rather upsetting to know that patients insist on using their own eggs and sperm even when advised against it, and it is not unheard of for couples to falsify their age to pursue ‘own-genetics’ (Klitzman, 2016). Making sure that patients fully understand the chances of success is also clearly important. Transparency of information is key: explaining to >40 years old woman that less than one out of 10 women gets a baby when using their own oocytes but three out of them succeed when using donated oocytes may be received more clearly than stating that egg donation increases the cumulative LBR for women aged over 40 years from under 10% to 30% per embryo transfer (Human Fertilisation and Embryology Authority, 2018).

Discussing with patients what would be a successful outcome for them could prove useful. This approach should include the potential harms and benefits of opting for both a child with missing genetic link(s) and for a child with full progeny, including a prognosis-based chance for success with cumulative success rates and time until live-born child. When the optimal outcome is to take a baby home, de-stigmatised use of donation for many would sit higher in the discussion than it currently seems to.

In patients with poor prognosis, asking them what the meaning of parenthood can be a good conversation starter. For many patients, the only contact they seem to have with hereditability is at the time of explaining that donors are matched in some traits, and hence genetics is emphasised without even discussing it. Explaining the meaning of genetics and heritability might lead to a more effective decision-making process. However, while it is important to share with patients that we are increasing our knowledge of the role of genetics at a very fast pace and that a cumulative number of diseases and traits are being identified through DNA testing, a counterbalance is necessary. Hereditability can explain the variation amongst traits but not causality. For example, we can explain to patients that height is a trait that has 90% of hereditability. If we take data from a poor village in an underdeveloped country 100 years ago, men were on average much shorter than the country’s present average, and still the hereditability was of 90%; sanitation and nutrition then raised the height of the next generations.

In addition, patients should be given the option to explore their preconceptions and share their fears about using gamete donation earlier in their treatment. The lack of high clarity information provision to patients from their GPs./fertility specialists about having a child versus an own-genes child may reflect the difficulty in broaching the subject of donor gametes as an option and anxiety about how to balance the conversation. We also need to acknowledge that physicians are not good at doing nothing (Watson, 1976) so may move towards the standard offer of ART.

We are all aware of the current shortage of donors in many countries. This should not mean that, because of a shortage, patients with poor prognosis can be under treatment with their own gametes while on a waiting list. Education on these issues might not only help ART patients in making better informed decisions but also raise the number of donors by including them in awareness campaigns.

Finally, we should note that we have not discussed religion within this context. The balance of religious groups and adherence is itself evolving (Hackett and McClendon, 2017), as this also leads to some countries considering donation illegal. The views of the couple/individual should always be taken and not assumed. Due consideration also needs to be given to religion in a local and global context alongside ancestry testing for the individuals and families concerned: in particular, where a donor may be regarded as the father of the child, after genetic testing, in a different judicial system outside that where treatment occurs, but where the donor or their family may at some stage be subject to the law of that other country.

## Conclusion

We believe that the wording used publicly and with patients must reflect and balance that a wish to become parents need not focus upon increasingly technical and potentially risky and unsuccessful measures to propagate one’s genes. In fact, an optimal medical approach should consider as the major decider the wish of the couple for parenthood and explain in a balanced manner the likelihood of this in the differing scenarios they face. However, the importance of carefully providing these choices at the start of the entire process is a key. As a couple begins a journey towards assisted conception, particularly when financial resources are limited, they need from the start to be able to consider whether they are aiming to replicate their genes and/or have children, where is the balance and what is most important to them. Only when we provide patients with the service of a clear and transparent discussion of these matters, we will really realise the true potential of our field, which may then be better considered as assisted families.

Key messages for current practice are as follows:

(i) A finding of reproductive difficulty is a surprise to most individuals. Information provided in these early stages of anxiety through medical contact helps form their future expectations. Early provision of balanced information with realistic discussions of rates of childlessness may help to better guide decisions.
(ii) Fertility clinics should make sure that patients have a clear idea of their success rates with different options, at regular intervals of time.
(iii) With the increasing options and advances aimed to assist genetic continuity in ART, there is a danger that individuals delay the process of becoming parents, potentially even contributing to childlessness.
(iv) Counselling is of paramount importance to every couple with a poor prognosis, not only for those who have already had repeated failures with their own gametes and are considering donation. Patients should be given the opportunity to expose their expectations and preconceptions about the impact of hereditability and environmental influences.
(v) Patients should be informed about the impossibility of guaranteeing anonymity.

Next steps for research are as follows:

(i) Analysis of ART outcomes would benefit from the results of LBR per patient and number of previous cycles with type of treatment, so that both reproductive healthcare professionals and patients could better support their decisions to move to donation.
(ii) Comparison studies of long-term health outcomes in donor-conceived children versus autologous cycles are needed to demystify the concerns of patients who have opted for donation regarding the health of the future children.
(iii) Randomised controlled trials are needed to test if getting a personalised assessment of success rates with decision-making approaches increases the patient’s understanding of prognostic information.
(iv) Research is needed to know if education on inheritability and attachment and exploration of fears regarding the use of gamete donation might lead to faster decisions (including the option for a childfree lifestyle), lower dropout rates and less involuntary childless individuals with a sustained child-wish.
(v) Further studies on the psychosocial outcomes in donor-conceived children, and their families and donors, versus autologous cycles are needed to further allay concerns of patients and some healthcare practitioners.
